# Relationship between Serum Vascular Endothelial Growth Factor Levels and Stages of Diabetic Retinopathy and Other Biomarkers

**DOI:** 10.1155/2020/8480193

**Published:** 2020-07-22

**Authors:** Nakhleh E. Abu-Yaghi, Nafez M. Abu Tarboush, Ala M. Abojaradeh, Amal S. Al-Akily, Esra'a M. Abdo, Laith O. Emoush

**Affiliations:** ^1^Department of Special Surgery, Ophthalmology Division, School of Medicine, The University of Jordan, Amman, Jordan; ^2^School of Medicine, The University of Jordan, Amman, Jordan; ^3^Department of Biochemistry and Physiology, School of Medicine, The University of Jordan, Amman, Jordan

## Abstract

**Aim:**

This study aims to measure serum vascular endothelial growth factor (VEGF) levels in a sample of Jordanian patients and to determine their relationship with the different stages of diabetic retinopathy. It also explores the correlation between VEGF concentrations and different biochemical and demographic findings.

**Materials and Methods:**

A total of 167 adults participated in the study. Participants were divided into two main categories: patients with diabetes mellitus (DM) type 2 without diabetic retinopathy (DR) (*N* = 62) and patients with DM type 2 affected by DR (*N* = 105). DR patients were further subclassified into nonproliferative (*N* = 41) and proliferative (*N* = 64). Basic laboratory tests were measured to correlate with VEGF levels. Irisin, a hormone linked to diabetic retinopathy was also measured and correlated with VEGF.

**Results:**

Serum VEGF was found to positively correlate with the severity of diabetic retinopathy. The means of VEGF serum concentrations were 60 pg/mL for controls, 133 pg/mL for nonproliferative DR patients, and 229 pg/mL for proliferative DR patients. We found a significant positive correlation with glycosylated hemoglobin (HbA1c), and a significant negative correlation with high-density lipoprotein (HDL) levels, age, and irisin.

**Conclusion:**

In this cohort of Jordanian diabetics, serum VEGF concentrations strongly correlated with the presence and stages of diabetic retinopathy, suggesting it as an appropriate indicator for diabetic retinopathy early detection and management in this society. VEGF levels also significantly correlated with HbA1c, HDL, and irisin levels. Further studies are encouraged to explore these relationships in other ethnic groups and with different diabetic complications.

## 1. Introduction

Diabetes mellitus (DM) type 2 is characterized by a chronic hyperglycemic state which eventually leads, if uncontrolled, to damage in specific organs including eyes, kidneys, and peripheral nerves [1]. Ophthalmic complications include diabetic retinopathy (DR), glaucoma, cataracts, corneal abnormalities, neuropathies, and iris neovascularization [2]. DR is the most common and feared ophthalmic complication due to DM and has become the leading cause of vision loss in working-age adults [3]. Signs of DR are present in one-third of DM type 2 patients at the time of diagnosis, and over 60% of patients with DM type 2 will develop DR after 20 years of disease onset [4].

Advanced DR occurs when fragile new blood vessels form on the surface of the retina over time [5]. These abnormal vessels can bleed or develop scar tissue causing significant loss of sight [6]. Accompanying scar tissue can contract and cause retinal detachment from underlying tissues, thus causing permanent loss of vision. DR is classified based on the growth of new vasculature in the retina into nonproliferative (NPDR) and proliferative (PDR) [6], with the latter being a more advanced stage and most commonly responsible for visual threatening complications [7].

Due to the high and rising rates of DR caused by DM type 2, it is essential to develop strategies to detect and manage this complication as early as possible. One of these strategies is the search for molecules that are associated with DR. Hence, extensive research was and is currently being conducted to investigate possible molecules linked to DR [8, 9].

Vascular endothelial growth factor (VEGF) has emerged as an important factor associated with DR development and an important prognostic indicator of the disease [10, 11]. VEGF is a 45-kDa homodimeric glycoprotein which belongs to a wide family of growth factors [12]. It is considered one of the main proteins that induce angiogenesis [13] and regulate vascular permeability by stimulating VEGFR-1 and VEGFR-2 receptors [14]. In DR, VEGF is produced by retinal cells to stimulate the development of new blood vessels adjacent to hypoxic areas [12, 15]. VEGF also promotes microaneurysm formation and has a role in increasing the permeability of the blood-retina barrier, stimulating the neoangiogenesis process in advanced DR [13, 16]. Anti-VEGFs have been developed to neutralize the intraocular VEGF of diseased eyes by intraocular injection [17]. Metformin, which is considered the first line therapy for DM type2, was found to decrease the phosphorylation of VEGFR-2 receptor, hence inhibiting the downstream pathway resulting in a decrease in the severity of neovascularization in advanced DR [18].

Few regional studies and many international studies have revealed a significant difference in serum VEGF levels between patients with DM type 2, with or without DR [19, 20]. A meta-analysis that was conducted in 2019 showed that serum VEGF levels correlate with the existence and severity of DR, therefore suggesting that serum levels of VEGF is a reliable indicator for DR evaluation and progression [21]. However, the relationship between VEGF serum levels and stages of DR has not been significantly explored in the Arab or Middle East regions and, up to our knowledge, was never explored in the Jordanian population where diabetes is prevalent [22].

Serum VEGF has also been studied in relation to other biochemical and demographic factors, such as glycosylated hemoglobin A1c (HbA1c), high-density lipoprotein (HDL), low-density lipoprotein (LDL), body mass index (BMI), total cholesterol (TC), triglycerides (TGs), and age without conclusive results [23–25]. Recently, DR patients have been investigated for possible association with a newly discovered myokine named “irisin” [26] that has been shown to be negatively associated with DM type 2 [27, 28]. Irisin induces browning of white adipose tissue thus protecting against weight gain and insulin resistance [29]. VEGF and irisin levels have never been correlated in the medical literature before.

Here, we aim to measure serum VEGF levels in a sample of Jordanian DM type 2 patients and determine the difference in VEGF levels among different stages of DR and investigate the correlation between VEGF concentrations and different biochemical and demographic findings including irisin.

## 2. Materials and Methods

### 2.1. Ethical Statement

This study has been conducted according to the Declaration of Helsinki (1964) and its amendments. The study has been approved by Jordan University Hospital (JUH) Institutional Review Board (IRB). Written informed consent has been obtained from all study participants.

### 2.2. Data Collection

A total of 167 adults participated in this study. Sample size was determined by power analysis and sample size software with *α* of 0.05 and power of 90%. Recruitment of participants and sample collection was from March to December 2016 at the Ophthalmology Clinic at JUH. Participants were divided into two main categories: patients with DM type 2 not affected by DR as a control group (*N* = 62) and patients with DM type 2 who are affected by DR (*N* = 105). DR patients were further subclassified into two categories: NPDR (*N* = 41) and PDR (*N* = 64). Whole blood samples were collected into plain tubes. Samples were centrifuged at 1,600 ×*g* for 15 min at 4°C. Serum has been kept at −80°C for a period less than a month.

### 2.3. Serum VEGF and Irisin Measurements

The quantitative measurements of VEGF in human serum samples were performed using a commercial enzyme linked immunosorbent assay (ELISA) kits for VEGF (CK-11550, SINNOWA Medical Science & Technology, Jiangsu, China) per the manufacturer's instructions. The absorbance from each sample was measured by a spectrophotometric microplate reader at a wavelength of 450 nm (Synergy™ HTX Multi-Mode Microplate Reader, Biotek, VT, USA). The sensitivity of the VEGF assay is typically less than 1 pg/mL and the linear range of the standard is 12.5–400 pg/mL. For irisin, the quantitative measurements in human serum samples have been performed using ELISA kit (EK-067-16, Phoenix Pharmaceuticals Inc., CA, USA) per manufacturer's instructions. Absorbance from each sample has been measured in duplicate by the same spectrophotometric microplate reader at wavelength of 450 nm. The sensitivity of the assay is 6.8 ng/mL and the linear range of the standard is 6.8–96.1 ng/mL.

### 2.4. Other Demographic and Biochemical Findings

Anthropometric measurements have been obtained using standard protocols and techniques. Body mass index (BMI) has been calculated as weight in kilograms divided by the square of height in meters (BMI = weight (kg)/(height (m))2) and used as a measure of obesity. Participants with BMI (kg/m^2^) <18.5, 18.5–24.5, 25–29.9, and ≥30 have been considered underweight, normal, overweight, and obese, respectively. Basic laboratory tests including HbA1c, HDL, LDL, TGs, and TC have been measured and recorded at the hospital laboratories.

### 2.5. Statistical Analysis

GraphPad PRISM 5 Statistical software and EXCEL Microsoft Office Professional Plus 2016 were used for statistical analyses. Continuous variables were expressed as mean ± SD. Associations of circulating VEGF levels with patients' laboratory findings and Irisin levels have been investigated. Unpaired *t*-test was used to compare two groups, one way singly analysis of variance (ANOVA) was used to compare more than two groups, and spearman correlation analysis was used to investigate associations. Probabilities of less than 0.05 were considered statistically significant.

## 3. Results

### 3.1. Demographic and Clinical Parameters

The demographic and clinical parameters of DR patients and controls are displayed in Table 1. There were 93 males and 74 females recruited in the study. Sixty-two patients had DM type 2 without DR and served as controls, while 105 patients had DM type 2 with DR including NPDR and PDR. DR patients were within the age group of controls. DR patients demonstrated higher and lower significant levels of HbA1c and irisin, respectively. Other clinical parameters were not significantly different between both groups.

### 3.2. VEGF Analysis of DR Patients and Controls

**Figure 1:**
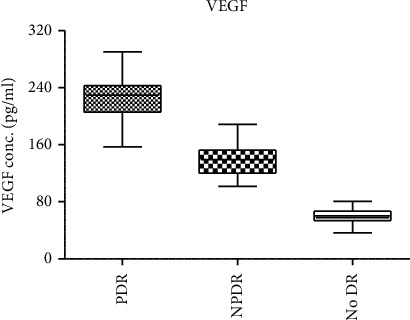
Mean concentration of VEGF hormone in serum samples of patients with DM type 2.

One hundred and sixty-seven blood samples were analyzed by ELISA for VEGF concentration (sixty-two samples of patients without DR, and 105 samples of patients with DR involvement). The means of VEGF serum concentrations for controls (*N* = 62) and DR participants (*N* = 105) were 60 ± 17.4 pg/mL and 192 ± 66.3 pg/mL, respectively. VEGF results were further analyzed for DR patients per their classification. NPDR patients (*N* = 41) had a mean of (133 ± 36.5 pg/mL), while PDR patients (*N* = 64) had a mean of (229 ± 52.0 pg/mL) (Figure 1). One-way single analysis of variance (ANOVA) revealed a statistically significant difference among the three groups (*p* < 0.001). Unpaired *t*-test demonstrated statistically significant differences among each two groups (*p* < 0.001).

VEGF: vascular endothelial growth factor. DR: diabetic retinopathy. PDR: proliferative diabetic retinopathy. NPDR: nonproliferative diabetic retinopathy. Mean concentration of VEGF ± SD is shown. The difference is statistically significant among the different groups of patients (*p* < 0.001). Unpaired *t*-test demonstrated statistically significant differences among each two groups (*p* < 0.001).

### 3.3. VEGF Correlation to Demographic and Clinical Parameters

**Figure 2:**
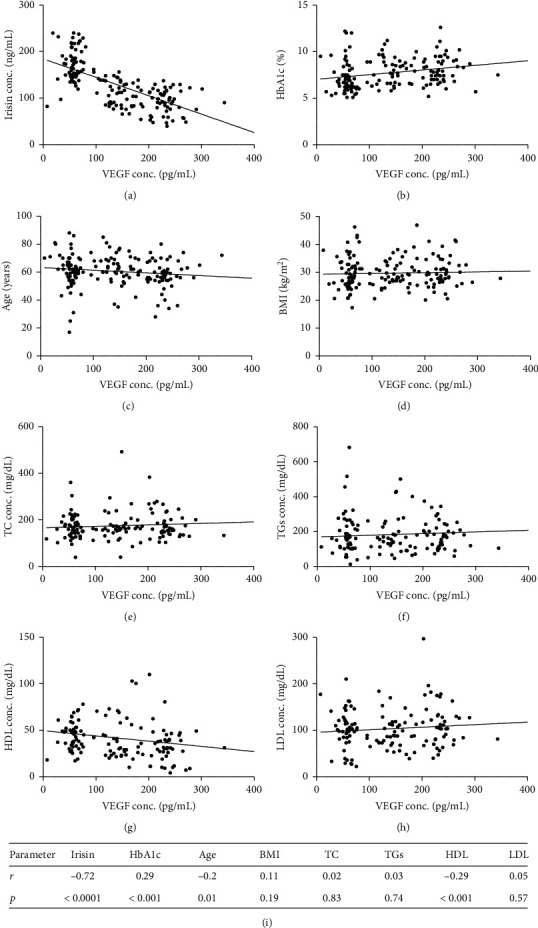
Correlation of VEGF serum level to the different clinical parameters tested for participants. VEGF: vascular endothelial growth factor. TC: total cholesterol. HbA1c: glycosylated hemoglobin. TGs: triglycerides. HDL: high-density lipoprotein. LDL: low-density lipoprotein. BMI: body mass index. *r* indicates spearman correlation coefficient and values of *p* less than 0.05 were considered significant.

We also analyzed the presence of a correlation between serum VEGF concentrations and biochemical findings (HbA1c, TC, TGs, HDL, LDL, BMI, and irisin). Spearman correlation analyses revealed significant positive correlation with HbA1c (*r* = 0.29, *p*=0.0002), and a significant negative correlation with age (*r* = −0.20, *p*=0.0109), HDL (*r* = −0.29, *p*=0.0007), and irisin (*r* = −0.72, *p* < 0.0001) levels. No significant correlation was found with TCs, TGs, BMI, or LDL levels (Figure 2).

## 4. Discussion

VEGF is a molecule that promotes angiogenesis and plays a critical role in the pathogenesis of DR [12, 14]. It is also one of the major indicators being studied for DR monitoring and its suppression in ocular tissues is a major target for DR treatment [17]. Studies show that VEGF serum levels in patients with DM type 2 are higher than healthy controls [30]. When injecting VEGF into animal eyes, changes similar to patients with DM type 2 or ischemic retinopathy were seen such as vessel tortuosity, retinal edema, intraretinal hemorrhages, and vascular proliferation [31]. It was also found that iris neovascularization was prevented upon inhibition of VEGF [32]. Studies on the vitreous and fibrovascular tissues of human eyes have shown higher levels of VEGF in eyes affected by PDR compared to NPDR affected eyes [33, 34].

Studies in the region considering VEGF levels are scarce. Two studies conducted in Egypt revealed a possible association between serum VEGF levels and diabetic retinal complications including a significant difference in serum VEGF levels between PDR and NPDR which lie in accordance with results presented here [25, 35]. Other reports in the Middle East including Saudi Arabia, Iraq, Iran, and Turkey [36–39] have only studied the relationship of VEGF levels and diabetes without differentiating between proliferative and nonproliferative DR. However, results obtained from those studies were also in line with results presented here where VEGF is higher in DM type 2 patients with DR compared to no-DR patients. Internationally, in spite of the conflicting results from different projects [40–43], most studies indicate a higher VEGF serum level in DM type 2 patients affected by DR. Nevertheless, variations in VEGF serum levels exist either regionally or internationally.

Our results demonstrate that serum VEGF levels were significantly higher in DM type 2 patients with DR compared to those with no DR. Furthermore, we found significantly higher levels of serum VEGF in patients with PDR compared to those with NPDR. These results are mirrored by reports from other parts of the world [21, 28, 43, 44]. Thus, consideration of VEGF levels as a suitable tool for risk assessment in DM type 2 patients regarding microangiopathic complications is an interesting concept despite the variation in VEGF levels amongst studies, which may reflect inherent demographic susceptibility.

This variation has been studied extensively in a recent meta-analysis that was conducted in 2019 and reviewed 29 studies with a total of 1805 DR (NPDR or PDR) patients and 1699 DM patients without DR [21]. The analysis had high heterogeneity among studies observed, but subgroup analyses and meta-regression analyses were used to adjust for potential confounders. They concluded that serum VEGF levels correlate with the existence and severity of DR, therefore suggesting that the serum VEGF level is a reliable indicator for DR evaluation and progression. Considering the VEGF level variation, the authors suggested that study location, design, and publication year of study may explain the heterogeneity in the different levels for serum VEGF among different studies [21]. We believe that our results add a much needed baseline pertaining to our community.

This study also uncovers a statistically significant correlation between VEGF levels and different biomarkers such as HbA1c, HDL, and Age. We found a significant positive correlation with HbA1c, a finding reported in previous studies where HbA1c was significantly correlated with the severity of DR [23]. This is extremely important for DM type 2 patients and should be signified by general practitioners, ophthalmologists, and other specialists dealing with diabetic patients. Serum VEGF had a negative correlation with HDL levels and age in our cohort. The same results regarding HDL were found in previous studies [24]. Considering age, controversial results in correlation to VEGF levels have been published and this can be explained by the age range of the population under study and the distribution of participants to the different disease progression groups [25].

Regarding irisin, we found a negative relationship between levels of VEGF and this myokine. Irisin is a hormone which has recently been investigated as a potential biomarker for DR and was found to have lower concentrations in patients with DR compared to DM type 2 without retinopathy [26, 28]. Irisin has also been found to behave as an anti-inflammatory agent that protects against DR through modulating the inflammatory molecule interleukin-17A [45]. Further, irisin was found to be lower in DM type 2 patients compared to healthy controls and decreases in level as the disease progresses with respect to ophthalmic complications (NPDR versus PDR) [26, 28]. VEGF level increases as retinopathy pathologically progresses; thus, it is rational for VEGF to be in negative correlation to irisin. The relationship between VEGF and irisin has never been examined in previous studies which makes this result vital for future studies.

The present study has the intrinsic limitation of being a case-control study where associations and differences are concluded, but causation is not investigated. Association of VEGF with other diabetic complications has not been explored in this project. Such relationships are expected to be intriguing as well. Also, measuring VEGF levels in samples of DM type 2 patients with and without DR involvement using multiple ELISA kits might explain the variation in serum VEGF levels in the literature and should be explored as a future direction. Up to our knowledge, this is the first study to document serum VEGF levels in the Jordanian population and establish a link with DR in such a cohort. Also, this is the first attempt to explore the relationship between VEGF and irisin.

## 5. Conclusion

We conclude that, in this cohort of Jordanian diabetics, serum VEGF levels strongly correlated with the presence and stages of DR, showcasing this biomarker as an appropriate indicator for DR early detection and management in this ethnic group. Serum VEGF concentrations were also found to be statistically correlated with some biochemical (HbA1c, HDL, and irisin) and demographic findings (age). Further studies are encouraged to explore these relationships in other societies and with other diabetic complications.

## Figures and Tables

**Table 1:** Demographics and clinical characteristics of DM type 2 patients included in the study. The table shows the comparison of means for each demographic and clinical finding for patients not affected (no DR) and affected by DR (NPDR and PDR).

Parameter	All DM type 2 participants		DR group	
N or (mean ± SD)	No DR group	DR group	NPDR group	PDR group
Males/females	29/33	64/41	27/14	37/27
Age (mean in years)	61 ± 11.1	60 ± 10.2	63 ± 10.9	58 ± 9.6
BMI (kg/m^2^)	29 ± 6.3	30 ± 5.3	30 ± 5.1	30 ± 5.6
HbA1c (%)	7.2 ± 1.6	8.1 ± 2.0^*∗*^	8.1 ± 1.8	8.1 ± 1.6
TC (mg/dL)	165 ± 45.8	178 ± 71.4	165 ± 54.4	186 ± 79.2
TGs (mg/dL)	178 ± 118.5	181 ± 112.2	167 ± 108.8	190 ± 112
HDL (mg/dL)	44 ± 13.4	40 ± 38.8	39 ± 20.5	40 ± 47.2
LDL (mg/dL)	101 ± 37.1	106 ± 49.0	96 ± 40.3	112 ± 52.8
Irisin (ng/mL)	174 ± 27.7	99 ± 27.5^*∗*^	114 ± 22.4	90 ± 25.6

^*∗*^ *p* values <0.05. DR: diabetic retinopathy. NPDR: nonproliferative diabetic retinopathy. PDR: proliferative diabetic retinopathy. BMI: body mass index. HbA1c: glycosylated hemoglobin. TC: total cholesterol. TGs: triglycerides. HDL: high-density lipoprotein. LDL: low-density lipoprotein.

## Data Availability

The datasets generated and/or analyzed during the current study are available from the corresponding author upon reasonable request.
